# Total numbers and in-hospital mortality of patients with myocardial infarction in Germany during the FIFA soccer world cup 2014

**DOI:** 10.1038/s41598-021-90582-z

**Published:** 2021-06-17

**Authors:** Karsten Keller, Lukas Hobohm, Volker H. Schmitt, Martin Engelhardt, Philip Wenzel, Felix Post, Thomas Münzel, Tommaso Gori, Birgit Friedmann-Bette

**Affiliations:** 1 Department of Cardiology, Cardiology I, University Medical Center Mainz (Johannes Gutenberg-University Mainz), Langenbeckstrasse 1, 55131 Mainz, Germany; 2 Medical Clinic VII, Department of Sports Medicine, University Hospital Heidelberg, Heidelberg, Germany; 3 Center for Thrombosis and Hemostasis (CTH), University Medical Center Mainz (Johannes Gutenberg-University Mainz), Mainz, Germany; 4 German Center for Cardiovascular Research (DZHK), Partner Site Rhine Main, Mainz, Germany; 5 Department for Orthopaedics, Trauma Surgery and Hand Surgery, Klinikum Osnabrück, Osnabrück, Germany; 6 Institute for Applied Training Science, Leipzig, Germany; 7 Department of Internal Medicine and Cardiology, Catholic Clinic Koblenz, Koblenz, Germany

**Keywords:** Cardiology, Health care, Risk factors

## Abstract

Environmental stress like important soccer events can induce excitation, stress and anger. We aimed to investigate (i) whether the FIFA soccer world cup (WC) 2014 and (ii) whether the soccer games of the German national team had an impact on total numbers and in-hospital mortality of patients with myocardial infarction (MI) in Germany. We analyzed data of MI inpatients of the German nationwide inpatient sample (2013–2015). Patients admitted due to MI during FIFA WC 2014 (12th June–13th July2014) were compared to those during the same period 2013 and 2015 (12th June–13th July). Total number of MI patients was higher during WC 2014 than in the comparison-period 2013 (18,479 vs.18,089, *P* < 0.001) and 2015 (18,479 vs.17,794, *P* < 0.001). WC was independently associated with higher MI numbers (2014 vs. 2013: OR 1.04 [95% CI 1.01–1.07]; 2014 vs. 2015: OR 1.07 [95% CI 1.04–1.10], *P* < 0.001). Patient characteristics and in-hospital mortality rate (8.3% vs. 8.3% vs. 8.4%) were similar during periods. In-hospital mortality rate was not affected by games of the German national team (8.9% vs. 8.1%, *P* = 0.110). However, we observed an increase regarding in-hospital mortality from 7.9 to 9.3% before to 12.0% at final-match-day. Number of hospital admissions due to MI in Germany was 3.7% higher during WC 2014 than during the same 31-day period 2015. While in-hospital mortality was not affected by the WC, the in-hospital mortality was highest at WC final.

## Introduction

Environmental stress affecting a large number of people can be induced by catastrophes such as earthquakes^1–3^, and hurricanes^4, 5^ as well as terror^6, 7^ or war^2, 8^, but also by large sporting events^9–12^ and is associated with cardiovascular diseases and their acute manifestations like myocardial infarction (MI)^1–5, 7–12^. Nevertheless, the association between important soccer events and acute cardiovascular illnesses remains controversial. Although a few studies support the hypothesis that large sporting events may induce higher rates of cardiovascular events^9–11, 13^ as well as higher short-term mortality^12–14^, other studies failed to confirm these findings^15–17^.

The German national soccer team is one of the best teams in the world winning the Fédération Internationale de Football Association (FIFA) world championship four times, last in the year 2014, and finishing in the third place in 2006 and 2010. On European level the German soccer team became second in the Union of European Football Associations (UEFA) European championship 2008 and semifinalist in the year 2012. Importantly for our study, soccer is the most popular sport in Germany. Overall, more than 34.5 million people watched the German victory on television at the FIFA soccer world cup (WC) in Brazil 2014.

Thus, as approximately half of the German citizens occupied itself with the big sporting event, we aimed to investigate (I) the impact of the FIFA WC 2014 on total numbers and in-hospital mortality of patients with MI in Germany and (II) whether the soccer games of the German national team influenced the number of admissions and in-hospital mortality during the FIFA WC 2014.

## Results

### Comparison of MI patients admitted during the FIFA world cup 2014 and during the comparison-periods 2013, 2014 and 2015

Overall, 71,844 patients were admitted for MI during the four periods, the FIFA WC 2014 from 12th June 2014 to 13th July 2014 and during the comparison periods from 12th June to 13th July 2013 as well as from 12th June to 13th July 2015 without soccer WC and during the additional comparison-period between 14th of July and 14th August 2014. The total number of MI patients was significantly higher during FIFA WC 2014 than in the comparison-period 2013 (18,479 vs. 18,089; *P* < 0.001; representing an increase of 2.1%) and the comparison period 2015 (18,479 vs. 17,794; *P* < 0.001; representing an increase of 3.7%) (Table 1). Additionally, total number of MI patients was also higher during the FIFA WC 2014 in comparison to the period between 14th of July to 14th August 2014 (18,479 vs. 17,482, *P* < 0.001; representing an increase of 5.4%).

**Table 1:** Patients’ characteristics of 54,362 patients admitted for MI during the World Cup 2014 from 12th June 2014 to 13th July 2014 and in the comparison periods from 12th June 2013 to 13th July 2013 as well as 12th June 2015 to 13th July 2015 without soccer World Cup.

Parameters	Comparison period from 12th June 2013 to 13th July 2013	*P* value for difference between 2013 and 2014	World Cup 2014 from 12th June 2014 to 13th July 2014	*P* value for difference between 2014 and 2015	Comparison period from 12th June 2015 to 13th July 2015
Number of hospitalizations due to myocardial infarction	18,089	**< 0.001**	18,479	**< 0.001**	17,794
Age (years)	72.0 (59.0–80.0)	0.423	72.0 (59.0–79.0)	0.828	71.00 (59.0–79.0)
Age ≥ 70 years	9,673 (53.5%)	0.070	9,707 (52.5%)	0.390	9,267 (52.1%)
Female sex^a^	6,112 (33.8%)	0.456	6,312 (34.2%)	0.246	5,975 (33.6%)
In-hospital stay (days)	7.90 ± 8.27	0.682	7.86 ± 7.86	**< 0.001**	7.62 ± 7.72
Obesity	1,459 (8.1%)	0.218	1,556 (8.4%)	0.291	1,444 (8.1%)
**Comorbidities**					
Cancer	339 (1.9%)	0.459	366 (2.0%)	0.523	336 (1.9%)
Peripheral artery disease	935 (5.2%)	**0.003**	1,086 (5.9%)	0.350	1,005 (5.6%)
Heart failure			6,630 (35.9%)	0.757	6,412 (36.0%)
Chronic obstructive pulmonary disease	1,308 (7.2%)	0.344	1,384 (7.5%)	0.292	1,385 (7.8%)
Essential arterial Hypertension	10,404 (57.5%)	0.449	10,556 (57.1%)	0.246	10,272 (57.7%)
Hyperlipidemia	8,055 (44.5%)	**0.001**	8,555 (46.3%)	**0.047**	8,053 (45.3%)
Atrial fibrillation/flutter	3,598 (19.9%)	0.401	3,611 (19.5%)	0.668	3,509 (19.7%)
Renal insufficiency with glomerular filtration rate < 60 ml/min/1,73 m^2^	2,812 (15.5%)	0.143	2,976 (16.1%)	0.359	2,803 (15.8%)
Diabetes mellitus	5,067 (28.0%)	0.159	5,299 (28.7%)	0.390	5,030 (28.3%)
Smoking	1,340 (7.4%)	0.608	1,395 (7.5%)	0.523	1,375 (7.7%)
**Treatment**					
Cardiac catheter	12,420 (68.7%)	**< 0.001**	13,215 (71.5%)	0.645	12,764 (71.7%)
Percutaneous coronary intervention	9,849 (54.4%)	**< 0.001**	10,546 (57.1%)	0.144	10,290 (57.8%)
Drug eluting stent implantation	7,140 (39.5%)	**< 0.001**	8,229 (44.5%)	**< 0.001**	8,894 (50.0%)
Bare metal stent implantation	2,092 (11.6%)	**< 0.001**	1,579 (8.5%)	**< 0.001**	726 (4.1%)
Bioresorbable vascular scaffolds	160 (0.9%)	**0.002**	225 (1.2%)	0.634	207 (1.2%)
Coronary bypass surgery	1,043 (5.8%)	0.648	1,045 (5.7%)	0.758	993 (5.6%)
Transfusion of blood constituents	1,673 (9.2%)	0.871	1,700 (9.2%)	**0.047**	1,531 (8.6%)
**In-hospital events**					
In-hospital death	1,505 (8.3%)	0.992	1,538 (8.3%)	0.892	1,488 (8.4%)
Recurrent myocardial infarction	61 (0.3%)	0.140	80 (0.4%)	0.616	71 (0.4%)
Shock	1,151 (6.4%)	0.050	1,270 (6.9%)	0.091	1,304 (7.2%)
Cardio-pulmonary resuscitation	1,019 (5.6%)	0.133	1,109 (6.0%)	0.589	1,044 (5.9%)
Stroke	216 (1.2%)	0.478	206 (1.1%)	0.557	187 (1.1%)
Deep venous thrombosis or thrombophlebitis	126 (0.7%)	**0.006**	88 (0.5%)	0.255	100 (0.6%)
Pulmonary embolism	71 (0.4%)	0.146	56 (0.3%)	0.264	66 (0.4%)

^a^Data about patients’ gender was available in 54,361 patients.

When analysing the total numbers of MI patients of the months June and July of the whole timeframe of the years 2011–2015, we identified no statistical differences between the years (*P* = 0.297) and no trend over time (β 96.9 [95% CI − 132.1 to 325.8], *P* = 0.358). Nevertheless, number of MI hospitalizations were highest in the year 2014 (Figure S1 in the supplementary material). During the FIFA WC 2014 from 12th June 2014 to 13th July 2014, no weekly differences regarding the total numbers of MI were detected (*P* = 0.434).

Patient characteristics were comparable between the groups. In brief, median age (2013: 72.0 (59.0–80.0) vs. 2014: 72.0 (59.0–79.0) vs. 2015: 71.00 (59.0–79.0) years) and proportion of female patients (2013: 33.8% vs. 2014: 34.2% vs. 2015: 33.6%) were similar between the three periods. Patients did not differ substantially for relevant cardio-vascular risk factors, diseases and comorbidities (Table 1).

In contrast, we identified some differences between the groups regarding interventional treatments. While the use of cardiac catheter (68.7% vs. 71.5%, *P* < 0.001) and percutaneous coronary intervention (54.4% vs. 57.1%, *P* < 0.001) increased from 2013 to 2014, the numbers were stable between 2014 and 2015. The total numbers of coronary artery bypass surgeries were comparable between the 3 periods, whereas the usage of drug eluting stent implantations increased and the use of bare metal stents decreased from 2013 to 2015 (Table 1). Transfusions of blood constituents were more often administered in 2014 in comparison to 2015 (9.2% vs. 8.6%, *P* = 0.047).

In-hospital mortality (2013: 8.3% vs. 2014: 8.3% vs. 2015: 8.4%) as well as recurrent MI (2013: 0.3% vs. 2014: 0.4% vs. 2015: 0.4%) and all other investigated adverse in-hospital events were comparable between the periods (Table 1). Additionally, also the in-hospital mortality rates during the FIFA WC 2014 and during the period between 14th of July to 14th August 2014 were similar (8.3% vs. 8.1%, *P* = 0.515).

The logistic regression models demonstrated an impact of the FIFA WC 2014 on the total numbers of admitted patients due to MI compared to the comparison-period 2013 (univariate regression: OR 1.06 [95% CI 1.03–1.09], *P* < 0.001; multivariate regression model: OR 1.04 [95% CI 1.01–1.07], *P* = 0.013) as well as 2015 (univariate regression: OR 1.06 [95% CI 1.03–1.09], *P* < 0.001; multivariate regression model: OR 1.07 [95% CI 1.04–1.10], *P* < 0.001). The FIFA WC 2014 was also associated with higher total numbers of admitted patients due to MI in comparison to the period between 14th of July to 14th August 2014 (univariate regression: OR 1.09 [95% CI 1.06–1.12], *P* < 0.001; multivariate regression model: OR 1.09 [95% CI 1.05–1.12], *P* < 0.001).

**Figure 1:**
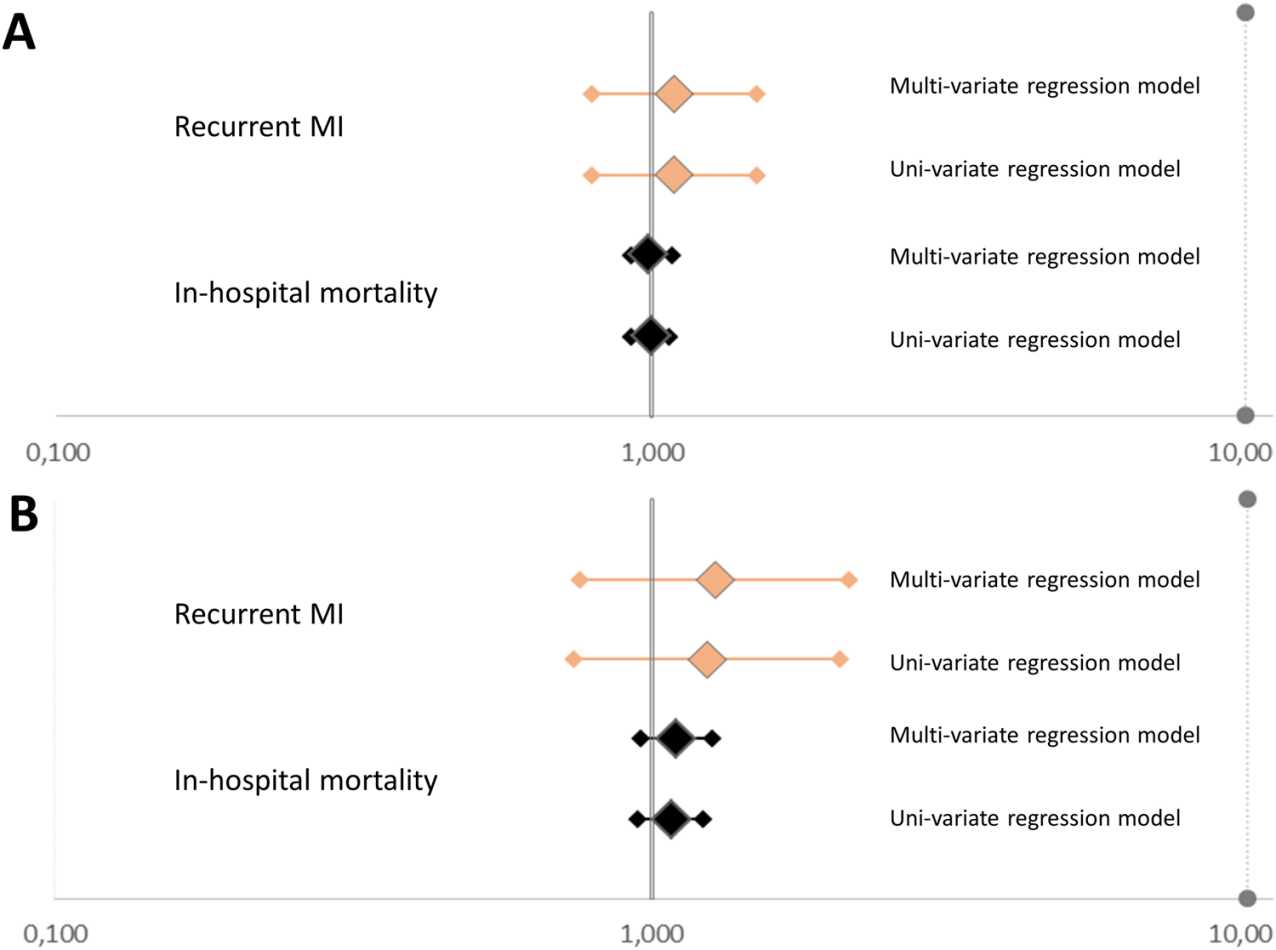
Impact on in-hospital mortality and recurrent MI. Logistic regression analyses for associations between the soccer world cup as well as the games of the German national team on recurrent MI and in-hospital mortality. (**A**) Impact of the soccer world cup 2014 on the outcomes recurrent MI and in-hospital mortality compared to the comparison-period 2015. (**B**) Impact of the German national team soccer games on the outcomes recurrent MI and in-hospital mortality compared with the other days of the world cup 2014.

In contrast, FIFA WC 2014 was not associated with an increase regarding in-hospital mortality. In-hospital mortality was not affected by the FIFA WC 2014 in comparison to the comparison period 2013 (univariate regression: OR 1.00 [95%CI 0.93-1.08; *P* = 0.992; multivariate regression model: OR 1.02 [95%CI 0.95-1.11], *P* = 0.567) and the comparison-period 2015 (univariate regression: OR 1.00 [95% CI 0.92–1.07], *P* = 0.892; multivariate regression model: OR 0.99 [95% CI 0.92–1.08], *P* = 0.872) (Fig. 1). The FIFA WC 2014 was independently associated with higher number of recurrent MI events in comparison to the comparison-period 2013 (univariate regression: OR 1.37 [95% CI 1.03–1.81], *P* = 0.030; multivariate regression model: OR 1.34 [95% CI 1.01–1.78], *P* = 0.039), but not associated with higher number of recurrent MI events in comparison to the comparison-period 2015 (univariate regression: OR 1.09 [95% CI 0.79–1.50], *P* = 0.616; multivariate regression model: OR 1.09 [95% CI 0.79–1.50], *P* = 0.614) (Fig. 1).

There were also no statistical differences regarding in-hospital mortality (*P* = 0.297) in the different years 2011–2015 when comparing the months June and July as well as no differences regarding mean temperature (*P* = 0.328) (Figure S1 in the supplementary material).

### Trends regarding total number of admitted MI patients, treatments and in-hospital mortality rate over the FIFA world cup 2014

**Figure 2:**
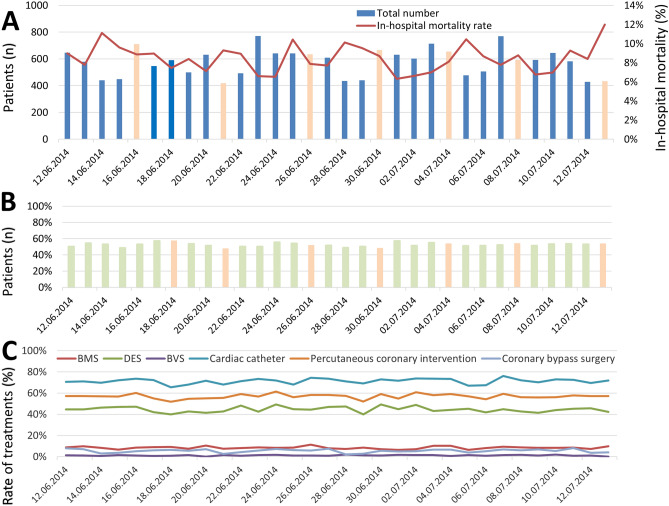
Temporal trends. (**A**) Absolute numbers of patients admitted due to MI events (bars) and in-hospital mortality rate (red line) across the soccer world cup 2014. The match days of German national team were colored in orange (16th June 2014 Germany vs. Portugal 4:0; 21st June 2014 Germany vs. Ghana 2:2; 26th June 2014 USA vs. Germany 0:1; round of 16: 30th June 2014 Germany vs. Algeria 2:1; quarterfinal: 04th July 2014 France vs. Germany 0:1; semifinal: 8th July 2014 Brazil vs. Germany 1:7; final: 13th July 2014 Germany vs. Argentina 1:0) and those days without German team-participation in blue. (**B**) Proportion of MI events in patients ≥ 70 years (green bars). (**C**) Proportion of MI events with different interventional and surgical treatments during the world cup 2014 (red line: bare metal stent (BMS) implantation; light green line: drug eluting stent (DES) implantation; violet line: bioresorbable vascular scaffolds (BVS) implantation; light blue line: cardiac catheter; orange line: percutaneous coronary intervention (PCI); dark blue line: coronary bypass surgery).

While the number of MI admissions throughout the WC remained unchanged (β 0.001 (95% CI − 0.033 to 0.035), *P* = 0.958), the in-hospital mortality rate decreased marginally over the whole FIFA WC 2014 period (β − 0.16 (95% CI − 0.23 to − 0.10), *P* < 0.001) (Fig. 2A).

Lowest number of admissions was seen during the second game of the preliminary round (21st June 2014; 419 admissions) with the soccer games between Argentina vs. Iran, Nigeria vs. Bosnia-Herzegovina and Germany vs. Ghana. In contrast, highest number of admissions during the FIFA WC 2014 was at the deciding games of the preliminary round (23rd June 2014) with 772 admissions due to MI with the games Cameroon vs. Brazil, Croatia vs. Mexico, Australia vs. Spain and Netherlands vs. Chile (Fig. 2A).

**Figure 3:**
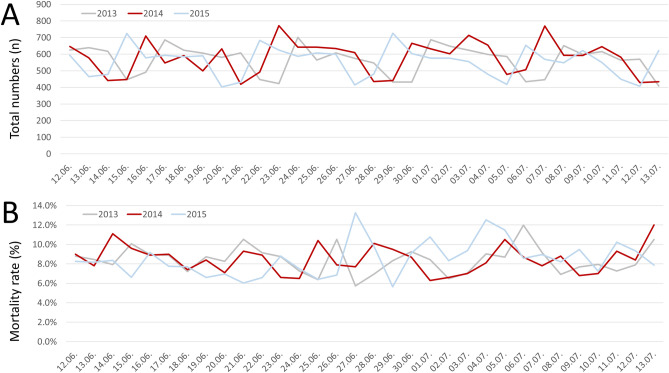
Absolute numbers of patients admitted due to MI events (**A**) and in-hospital mortality rates (**B**) across the observational periods 2013, 2014 and 2015. The data of the year 2013 is visible as grey line, 2014 as red line and the 2015 as blue line.

Similarly, the comparison periods of the years 2013 and 2015 showed also an up and down of the daily admission number due to MI over the observational periods (Fig. 3).

Interestingly, the in-hospital mortality was highest at the final match between Germany vs. Argentina with 12.0%. Lowest rate of in-hospital death was found at the 1st July 2014 with 6.3% and the matches Argentina vs. Switzerland and Belgium vs. USA (Fig. 2A). Highest total number of in-hospital death was 144 deaths at the final match (Figure S2 in the supplementary material). The mortality rate at days with lower admission rate (< 600 admissions with MI per day) versus those days with higher admission rate (≥ 600 patients) were not statistically different (*P* = 0.406).

We detected no trend regarding the proportion of patients aged ≥ 70 years as well as interventional treatments for MI over the observational FIFA WC 2014 period (Fig. 2B,C).

### Impact of the games of the German national team on admissions due to MI, in-hospital mortality and treatments

During the FIFA WC 2014, the total number of admitted MI patients (634 (434–666) vs. 591 (485–642), *P* = 0.624) as well as the in-hospital mortality rate (8.9% vs. 8.1%, *P* = 0.110) were not significantly affected by the games of the German national team (Table 2). Additionally, we analysed whether the timepoint of the first German goal in the matches of the German national team had an influence on the admission of patients with MI. In three of the seven games the first goal of the German national team was in the first half and respectively in the first 12 min of the matches. The timepoint of the first goal of the German national team had no influence on admissions of patients with acute MI (*P* = 0.321).

**Table 2:** Baseline characteristics of 18,479 patients admitted for MI during the World Cup 2014 from 12th June 2014 to 13th July 2014 stratified for soccer games of the German national soccer team versus the other days of world cup.

Parameters	Games of the German national soccer team (n = 4,110; 22.2%)	Period of the World Cup without games of the German national soccer team (n = 14,369; 77.8%)	*P* value
Age ≥ 70 years	2,130 (51.8%)	7,577 (52.7%)	0.305
**Treatment**			
Cardiac catheter	2,985 (72.6%)	10,230 (71.2%)	0.073
Percutaneous coronary intervention	2390 (58.2%)	8,156 (56.8%)	0.112
Drug eluting stent implantation	1,847 (44.9%)	6,382 (44.4%)	0.551
Bare metal stent implantation	372 (9.1%)	1,207 (8.4%)	0.188
Bioresorbable vascular scaffolds	45 (1.1%)	180 (1.3%)	0.416
Coronary bypass surgery	218 (5.3%)	827 (5.8%)	0.269
**In-hospital events**			
In-hospital death	367 (8.9%)	1,171 (8.1%)	0.110
Recurrent myocardial infarction	22 (0.54%)	58 (0.40%)	0.257

The performed interventional treatment rates for MI did not differ between the match days of the German national team vs. other days of the WC period.

These results were confirmed in the logistic regression models, showing no association between games of the German national team and recurrent MI events as well as mortality rate (Fig. 1B).

**Figure 4:**
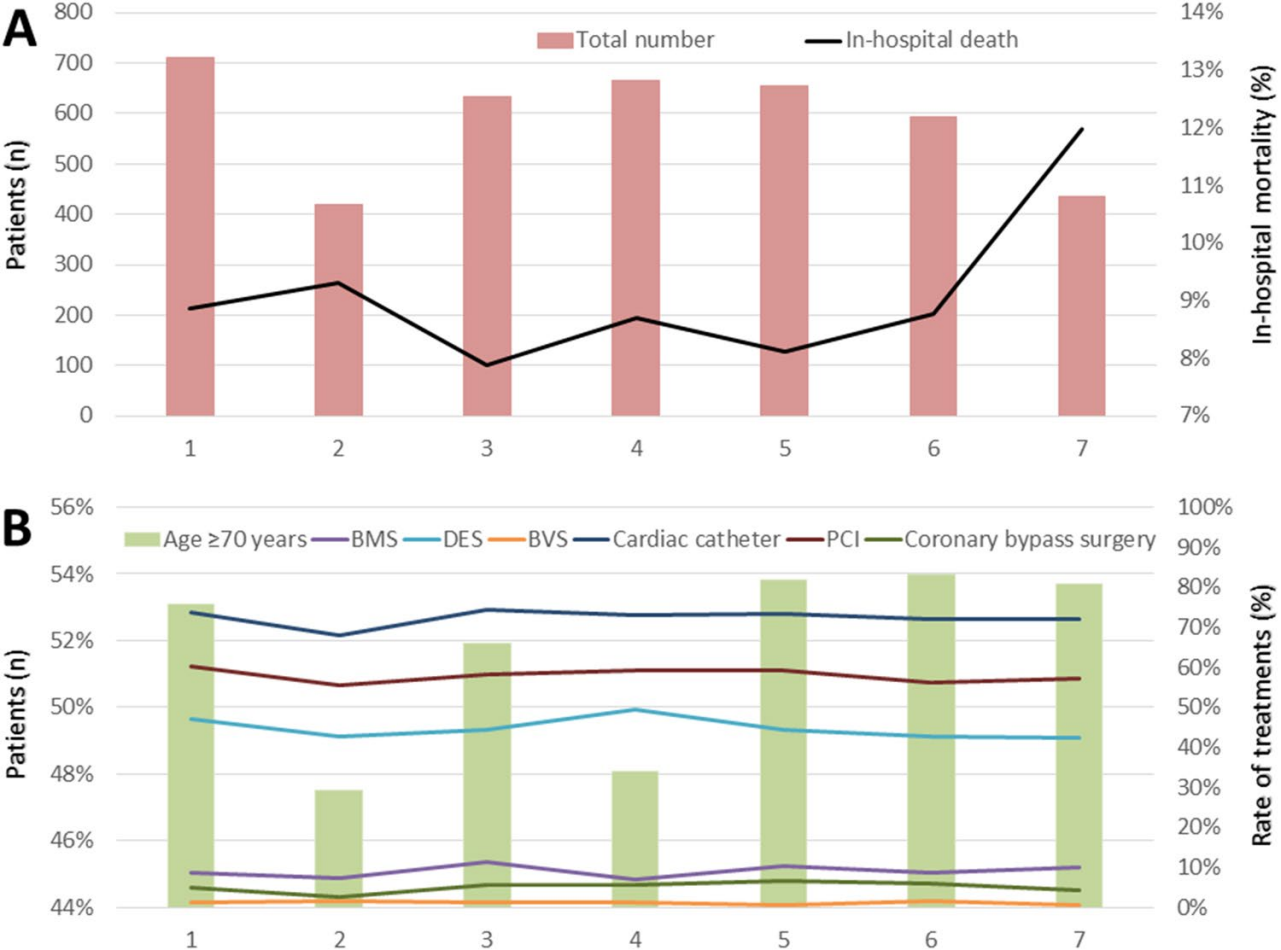
German soccer games during the FIFA world cup 2014. (**A**) Absolute numbers of patients admitted due to MI events (red bars) and in-hospital mortality rate (black line) across the German soccer games during the world cup 2014. (**B**) Proportion of MI events in patients ≥ 70 years (green bars) and different interventional and surgical treatments across the German soccer games during the world cup 2014 (violet line: bare metal stent (BMS) implantation; light blue line: drug eluting stent (DES) implantation; yellow line: bioresorbable vascular scaffolds (BVS) implantation; dark blue line: cardiac catheter; red line: percutaneous coronary intervention (PCI); green line: coronary bypass surgery).

However, we observed an increase of the in-hospital mortality rate from the match days before the final ranging between 7.9% and 9.3% to 12.0% at the final match day (Fig. 4A).

While the last three games of the German national soccer team (quarter-final, semi-final and final) were accompanied by the highest rate of MI patients ≥ 70 years, the interventional und surgical treatments for patients hospitalized for MI did not changed during these games (Fig. 4B).

The German nationwide inpatient sample showed no significant increase of the total number of MI events over the match days of the German national team during the FIFA WC 2014 (β − 0.006 (95% CI − 0.026 to 0.015), *P* = 0.502) (Fig. 4A). In addition, no statistical uptrend of in-hospital death during the WC from game one against Portugal to the WC final against Argentina in the linear regression analysis could be detected (β − 0.0003 (95% CI − 0.0009 to 0.0004), *P* = 0.416) (over the match days with participation of the German national team). Nevertheless, we observed highest mortality rate at the day of the FIFA WC final 2014, as already mentioned above (Figs. 4A and 2A).

## Discussion

Large sporting events potentially increase the occurrence of cardiovascular events^9–11, 13^ as well as related mortality^12–14^, but the results are not consistent across studies^15–17^. Soccer is the most popular sport in Germany and more than 34.5 million people watched the German victory on television at the FIFA WC 2014 in Brazil. Thus, we aimed to investigate both, (I) the impact of the WC 2014 on total numbers and in-hospital mortality of patients with MI in Germany and (II) whether the soccer games of the German national team influenced the number of admissions and mortality during the WC 2014. We focused on the strong outcome MI and decided not to investigate an impact of the WC on angina pectoris since angina pectoris is a weaker endpoint in comparison to the well-defined MI.

The main results of our study could be summarized as follows:

(I) The number of admissions due to MI in Germany was 3.7% higher during the FIFA WC 2014 than during the same 31-days period 2015 and 2.1% higher during the FIFA WC 2014 than during the same 31-days period 2013.
(II) In-hospital mortality of MI was not affected by the FIFA WC 2014 (compared to the 31-days comparison-periods 2013, 2014 and 2015).
(III) Admissions of MI patients as well as the in-hospital mortality during the FIFA WC 2014 were not influenced by the games of the German national team.
(IV) Nevertheless, highest in-hospital mortality of the whole WC period was observed at the FIFA WC final 2014 at the game Germany vs. Argentina (1:0).

Identification of potent triggers in the pathogenesis of MI are of outstanding importance^18^. Worldwide, ischemic heart disease with its acute manifestation MI is the single most common cause of death with a still increasing frequency^19^. It accounts for approximately 20% of all deaths in Europe^19^ and the USA^20^.

MI events are usually based on atherosclerotic stenosis of the coronary vessels, atherosclerotic plaques with plaque rupture leading to coronary thrombus formation resulting in myocardial hypoperfusion, inadequate myocardial oxygen supply, myocardial cell damage and myocardial cell death^21^. Besides these internal factors, which are directly influenced by typical cardiovascular risk factors such as smoking, family history of MI, adverse lipid profiles, and elevated blood pressure^21, 22^, MI is often preceded by specific triggers, which can include common activities such as physical exertion, alcohol consumption and heavy meals, air pollution but also stressful events^23–25^. In this context, it is well known, that the onset of MI follows a circadian and seasonal periodicity^26–33^. Beyond the seasonal and circadian variation, an increase of cardiovascular events was observed on Christmas and New Year’s Day as well as returning to work at Mondays which were associated with increased mental stress, respectively^18^. Thus, we compared in the present study the temperature during the investigated months (June and July) of the years 2011–2015 and found no significant difference between the investigated years for Germany. In addition, it has to be mentioned that the government did not change and the socioeconomic and political status of Germany were stable during the observational period. The German federal elections (Bundestagswahlen) were in the years 2013 and 2017 and the chancellor did not change over the whole observational period. Furthermore, we detected no influence of the weekdays on the admission rate during the FIFA WC 2014.

While 48% of the MI patients reported clinical triggers, moderate to heavy physical exertion was seen in 23% and emotional stress and upset in 18% of the MI patients^18^. Mental stress according to tension, sadness, frustration and anxiety increases the risk of myocardial ischemia^18, 24^. Although large sporting events are substantially less dramatic events than environmental catastrophes^1–5, 7–12^, studies have shown that these events could also affect the occurrence of cardiovascular diseases^9–11, 13, 17^ as well as related mortality^12–14^.

### Total admissions for MI patients

Our study results show a strong and substantial increase in the total numbers of MI during the soccer WC 2014 compared to the same 31-days comparison-periods 2013 and 2015. This finding is in accordance with previous studies about European soccer matches influencing cardiovascular events^9, 10, 13–15, 18, 34, 35^. In contrast to our study, Wilbert-Lampen et al. identified a significant increase of MI incidence in the German population during the FIFA WC 2006 exclusively at match days of the German national team compared to later and earlier years, but not an increased occurrence during the whole soccer WC (games without German team participation were not accompanied by significant increase regarding the occurrence of MI)^9^. The increase during the matches with German participation was more pronounced in men than in women^9^. In contrast, we could not confirm a higher admission rate of MI patients at match days with games of the German national team compared to those WC days without, although our sample was 8.5-times larger than that of Wilbert-Lampen et al., who investigated the number of cardiovascular emergencies in different emergency departments in Bavaria^9^. Nevertheless, differences in the studied periods might explain the controversies, since we looked for MI events at the day of the soccer game, other studies searched for affections of the game on MI events of both, the game day as well as the two days after the match^17^.

In addition, it has been reported that particularly, shoot-outs and defeats were associated with increased admissions^9, 10, 14^. Carroll et al. reported that the risk of admission due to MI was increased by 25% in England on the day England lost to Argentina in a penalty shoot-out and the following 2 days of the FIFA WC 1998^10^. In contrast, Gebhard et al. reported higher MI rates after victories of the Montreal Ice Hockey team^11^. Since the German team was not defeated at the FIFA WC 2014 and won the championship, we were not able to distinguish between match days with defeats and wins. However, our study demonstrated in accordance with most studies that WC soccer events are potent triggers of MI that should not be underestimated.

Pathophysiological, it has been hypothesized, that emitted stress hormones might directly affect endothelial, monocytic and platelet functions^17, 36^. Stress resulting in increased sympathetic nervous activity and coagulability contributes to an increased risk for transformation of a non-vulnerable atherosclerotic plaque into a plaque, which is susceptible for disruption accompanied by thrombogenic stimulus and can induce further vasoconstriction as well as increased coagulability, resulting in acute coronary syndrome including MI^14, 17^. This was supported by a study showing that watching the FIFA WC 2010 between Spain and the Netherlands, Spanish fans had significant higher levels of testosterone and cortisol compared with control days^17, 37^ and by another study showing changes in endothelial function in healthy volunteers during the FIFA soccer WC 2010^36^.

Nevertheless, Niederseer et al. observed that watching the FIFA WC 2006 was not associated with any changes regarding incidence of cardiac events in Bavaria^16^ and Aboa-Eboule et al. reported even a lower incidence of stroke events during the UEFA European soccer Championships from 1986–2006 in the city of Dijon^17^.

Regarding treatments some temporal trends/changes require attention: The treatment rate of BMS decreased from 2013 to 2015, while the use of DES increased substantially from 2013 over 2014 to the year 2015. This is an expected finding, since the recommendations of the guidelines changed over time towards the recommendation to use predominantly DES and BMS only in selected patients^19, 38–41^.

### In-hospital mortality of MI patients

Regarding in-hospital mortality, our study demonstrated that the in-hospital mortality of MI was not affected by the FIFA WC 2014 compared to the 31-days comparison-periods 2013, 2014 and 2015. This might not be surprising since most studies demonstrated that especially defeats of the favored team were accompanied by increased mortality^12, 14^.

While Witte et al. reported an increase regarding the mortality rate of individuals with stroke and coronary heart disease related to the Dutch soccer quarterfinal defeat against France in the shoot-out in males, but not in females at the UEFA European soccer championships 1996^14^, Toubiana et al. observed no increase in France caused by the same match, which might be attributed to the win of the French soccer team^15^.

In contrast, Katz et al. showed a 63% increase of sudden cardiac death in Switzerland during the FIFA WC 2002. Although the increase was more pronounced in men, the incline was also in women significantly visible^13^. Once again, since the German team was not defeated and won the FIFA WC 2014, we were not able to identify a defeat as a trigger of increased mortality. Nevertheless, the highest mortality rate and as well the maximum of total numbers of in-hospital deaths were observed at the final match, which might indicate for a high stress level of the German soccer fans at the very exiting final game of Germany against Argentina, which was won not before the extension time with 1:0. Although, most peaks of the in-hospital mortality rate during the FIFA WC 2014 in the timeframe before the final match were at days with lower number of admissions for MI and the total number of deceased patients were comparable to the other days of the WC, in contrast, at the final match both, the in-hospital mortality rate as well as the total number of deceased patients were at their maximum during the FIFA WC 2014. Thus, the matches before the final did not affect the in-hospital mortality significantly, while the final match was accompanied by a substantial increase of in-hospital mortality.

## Limitations

We have to report some limitations regarding our study that require consideration: Firstly, one major limitation is that the mentioned study results are based on ICD discharge codes, which might result in incomplete dataset due to under-reporting or under-coding. Secondly, clinical data like information about troponins, echocardiograms or concomitant medications are not available and therefore missing for additional analyses. Thirdly, with the data of the German nationwide inpatients sample reliable door-to-device or door-to-balloon time could not be calculated. Thus, the focus of the present study was on clear and strong endpoints such as in-hospital death and also recurrent MI which are very unlikely to be miscoded or not-coded.

## Conclusions

Watching the FIFA WC 2014 was a trigger of the occurrence of MI. While the number of admissions due to MI in Germany was 3.7% higher during the FIFA WC 2014 than during the comparison-period 2015, the in-hospital mortality of MI was not affected by the WC. Nevertheless, the final match of Germany vs. Argentina with a scant victory of Germany was accompanied by highest in-hospital mortality throughout the WC period. Our data may help to find better ways of planning hospital capacities, which is essential for delivering sufficient capacity at the right time point to meet future enormous health-care challenges.

## Methods

### Data source

The statistical data analyses were performed on our behalf by the Research Data Center (RDC) of the Federal Statistical Office and the Statistical Offices of the federal states (source: RDC of the Federal Statistical Office and the Statistical Offices of the federal states, DRG Statistics 2005–2017, and own calculations), in Wiesbaden (Germany). The aggregated statistic data were provided from the RDC to us on the basis of SPSS codes (SPSS Software, version 20.0, SPSS Inc., Chicago, Illinois), which we supplied to the RDC^33, 42^. For this analysis, we selected all patients admitted due to MI during the FIFA WC 2014 between 12th June and 13th July 2014 and during the comparison-periods of the years 2013 and 2015 between 12th June and 13th July 2015/2013 as well as between 14th of July to 14th August 2014.

### Diagnoses, procedural codes, and definitions

Based on a diagnosis- and procedure-related remuneration system in Germany (German Diagnosis Related Groups [G-DRG] system), it is mandatory for all hospitals to transfer the coded patient data of each patient on diagnoses, (coexisting) conditions, and procedures to the Institute for the Hospital Remuneration System in order to get their remuneration^42, 43^.

Diagnoses are coded according to the International Classification of Diseases and Related Health Problems, 10th Revision with German Modification (ICD-10-GM) and surgical, diagnostic or interventional procedures according to the German Procedure Classification (OPS, surgery and procedures codes [Operationen- und Prozedurenschlüssel]). Thus, we were able to identify all patients admitted for MI (ICD codes I21 and I22) during the WC period and the comparison-period^33^.

### Study outcomes

The outcomes of this study were number of admitted MI patients, death of all causes during the hospital stay (in-hospital death) and recurrent MI (ICD code I22), which was defined as recurrent MI during the first 28 days after a previous MI.

### Ethical aspects

Since this study did not involve direct access by the investigators to data of individual patients, approval by an ethics committee and informed consent were not required, in accordance with German law.

### Statistical methods

Descriptive statistics for relevant comparisons of MI patients admitted during the FIFA WC 2014 and those admitted during the comparison-periods 2013, 2014 and 2015 as well as MI patients admitted during the FIFA WC 2014 on match days of the German national soccer team and those on match days without German team-participation are provided as median and interquartile range (IQR), or as absolute numbers and corresponding percentages. Continuous variables were tested using the Mann–Whitney-U test and categorical variables were computed with Fisher’s exact or chi^2^ test, as appropriate.

Temporal trends regarding total numbers of MI, interventional treatments and in-hospital mortality rate were analysed over the period of the FIFA WC 2014 and linear regressions were used to test for significant increase/decrease. The Results were presented as Beta (β) and corresponding 95% confidence intervals (CI).

Univariate and multivariate logistic regression models were analysed to investigate the impact of the FIFA WC 2014 as well as the impact of the match days with participation of the German national team on the study outcomes total numbers of MI patients, recurrent MI and in-hospital mortality. The Results were presented as Odds Ratios (OR) and corresponding 95% CI. Multi-variate logistic regression model, testing the independence of predictors for in-hospital outcomes, included the following parameters for adjustment: age, sex, active cancer, coronary artery disease, heart failure, chronic obstructive pulmonary disease, arterial hypertension, hyperlipidaemia, smoking, diabetes mellitus, atrial fibrillation/flutter (ICD code I48), renal insufficiency (comprised diagnosis of chronic renal insufficiency stages 3 to 5 with glomerular filtration rate < 60 ml/min/1,73 m^2^), cardiac catheter, percutaneous coronary intervention, and coronary artery bypass surgery.

The software SPSS (version 20.0; SPSS Inc., Chicago, Illinois) was used for computerised analysis. *P* values of < 0.05 (two-sided) were considered to be statistically significant.

## Supplementary Information

Supplementary Information.

## Abbreviations

CI: Confidence interval
FIFA: Fédération Internationale de Football Association
ICD: International Classification of Disease
IQR: Interquartile range
MI: Myocardial infarction
OPS: Surgery and interventional procedure keys (Operationen- und Prozedurenschlüssel)
OR: Odds ratio
RDC: Research Data Center
UEFA: Union of European Football Associations
