# Supplementary material for: Total numbers and in-hospital mortality of patients with myocardial infarction in Germany during the FIFA soccer world cup 2014
Source: Sci Rep. 2021 Jun 17;11:11330. doi: 10.1038/s41598-021-90582-z (PMC8211804; doi:10.1038/s41598-021-90582-z)

**Supplementary Material**

**Results**

**Figure S1**: Temporal trends in Myocardial infarction, mortality rate and temperature

A: Temporal trends in total numbers of myocardial infarction (blue bars) and in-hospital mortality rate (red line) stratified for the months June and July 2011-2015

B: Temporal trend in mean temperature (yellow line) stratified for the months June and July 2011-2015


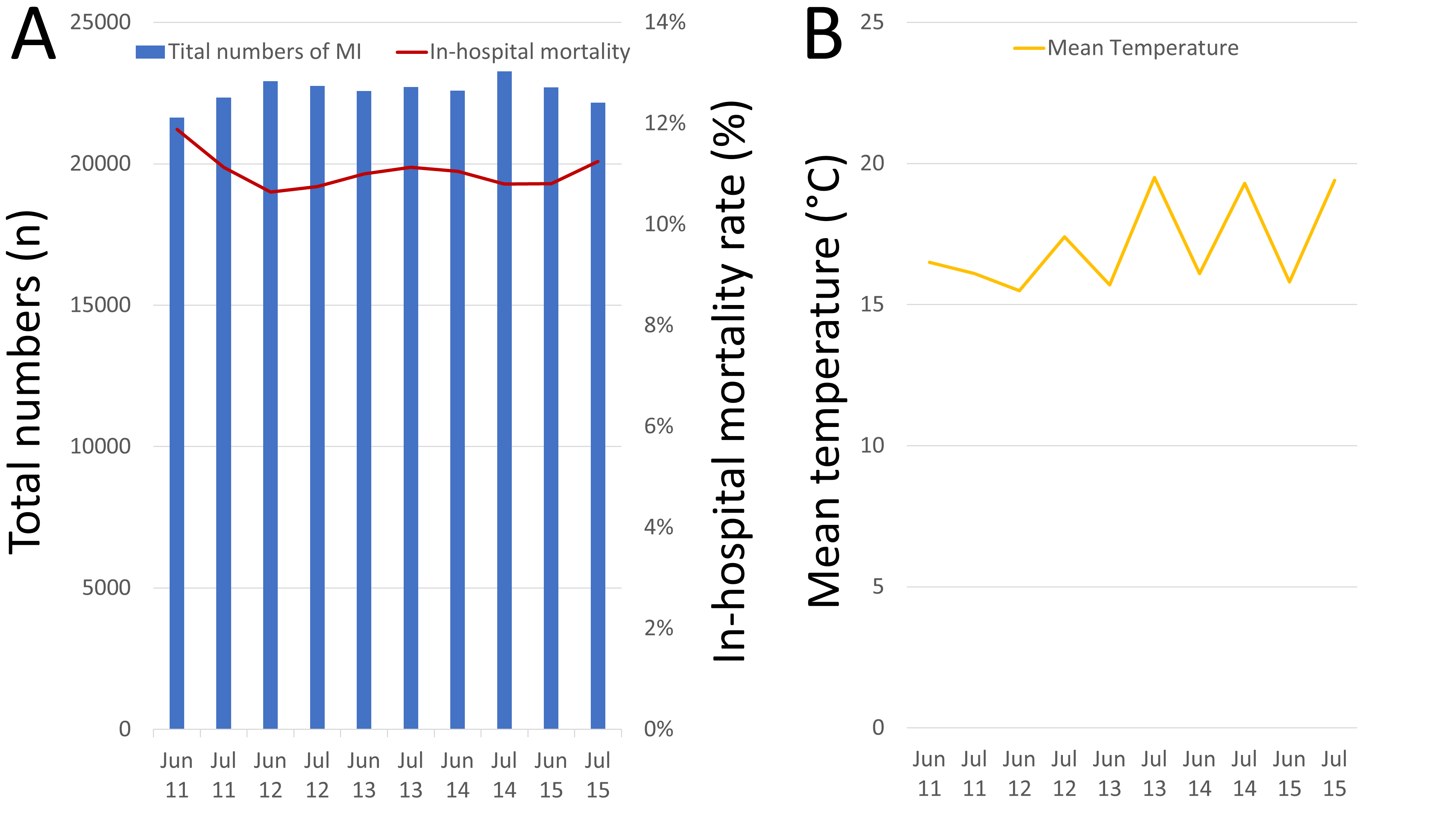


**Figure S2**: Temporal trends regarding the in-hospital deaths of patients with myocardial infarction during the FIFA WC 2014 from 12^th^ June to 13^th^ July 2014


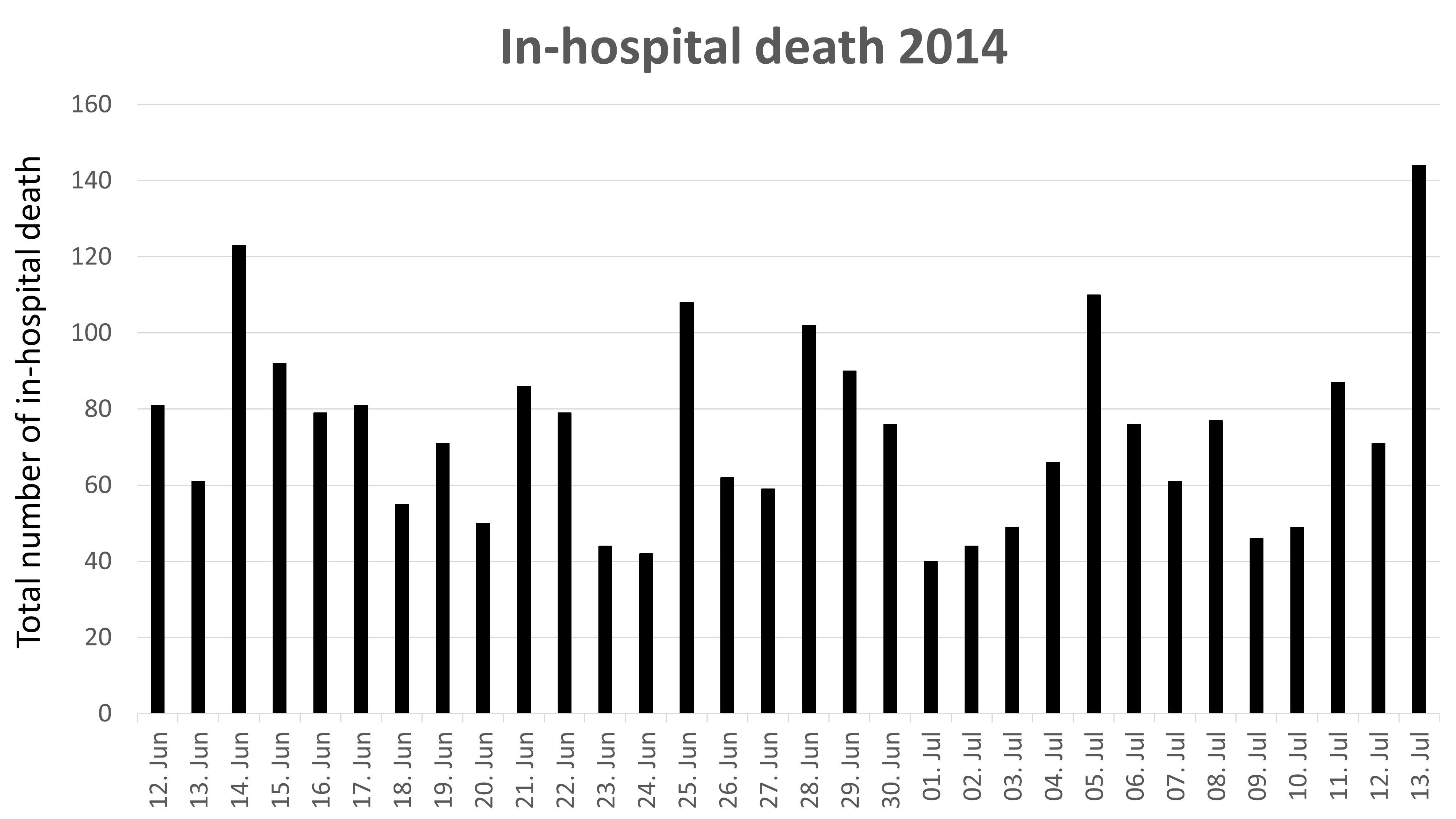

Supplement: Supplementary file 1 — Supplementary Information. [file 41598_2021_90582_MOESM1_ESM.docx]
